# Incidence and Diversity of Antimicrobial Multidrug Resistance Profiles of Uropathogenic Bacteria

**DOI:** 10.1155/2015/354084

**Published:** 2015-03-05

**Authors:** Inês Linhares, Teresa Raposo, António Rodrigues, Adelaide Almeida

**Affiliations:** ^1^Department of Biology and CESAM, University of Aveiro, 3810-193 Aveiro, Portugal; ^2^Clinical Analysis Laboratory Avelab, Rua Cerâmica do Vouga, 3800-011 Aveiro, Portugal

## Abstract

The aim of this study was to assess the most frequent multidrug resistant (MDR) profiles of the main bacteria implicated in community-acquired urinary tract infections (UTI). Only the MDR profiles observed in, at least, 5% of the MDR isolates were considered. A quarter of the bacteria were MDR and the most common MDR profile, including resistance to penicillins, quinolones, and sulfonamides (antibiotics with different mechanisms of action, all mainly recommended by the European Association of Urology for empirical therapy of uncomplicated UTI), was observed, alone or in association with resistance to other antimicrobial classes, in the main bacteria implicated in UTI. The penicillin class was included in all the frequent MDR profiles observed in the ten main bacteria and was the antibiotic with the highest prescription during the study period. The sulfonamides class, included in five of the six more frequent MDR profiles, was avoided between 2000 and 2009. The results suggest that the high MDR percentage and the high diversity of MDR profiles result from a high prescription of antibiotics but also from antibiotic-resistant genes transmitted with other resistance determinants on mobile genetic elements and that the UTI standard treatment guidelines must be adjusted for the community of Aveiro District.

## 1. Introduction

Multidrug resistant (MDR) bacteria are more usually associated with nosocomial infections. However, their emergence at the community level has increased, making the infections treatment more difficult, namely, the most common ones, such as the urinary tract infection (UTI). Uncomplicated cystitis (infection of bladder) in women is the most common UTI and according to the European Association of Urology (2013) is defined as the growth of a single pathogen of >10^3^ colony-forming units mL^−1^ from properly collected mid-stream urine [1]. Some studies performed at community level showed that MDR bacterial percentage observed among the most prevalent bacteria involved in the community-acquired UTI,* Escherichia coli*, varied between 38 and 54% [2, 3].

The increasing spread of bacterial resistance to antimicrobials at the community setting is promoted by several factors. The overuse and misuse of antimicrobials in human medicine, in veterinary and in agriculture represent some of the behaviours responsible for selective pressure which enables the selection and spread of clones that carry antibiotic-resistance genes [4]. Measures to prevent and control the increase of antimicrobial resistance as well as the dissemination of resistance genes are crucial. One of these measures is the reduction of antibiotic consumption by the implementation of norms for its rational use.

Resistance to antibiotics occurs classically as a result of drug modification, target alteration, and reduced accumulation owing to decreased permeability and/or increased efflux. It may be an innate feature of a microorganism or may result from mutation or acquisition of exogenous resistance genes [5]. The acquisition of resistant genes has been well described in the literature [6, 7] and it is particularly important because acquisition regularly might confer cross- or coresistance [7–9] which may turn bacteria MDR to specific antibiotics even when these antibiotics are not frequently prescribed or had even been abolished.

The 2013 guidelines of the European Association of Urology (EAU) recommend for empirical treatment of acute uncomplicated cystitis TMP-SMX (if the local resistance is less than 20% for* E. coli*), nitrofurantoin, fosfomycin trometamol and pivmecillinam (as first-line therapy). Fluoroquinolones may be used only as alternative therapy and their use must be avoided to treat uncomplicated cystitis whenever possible [1]. According to the 2009 EAU guidelines, however, fluoroquinolones were recommended as first-line empirical therapy [1, 10].

This study aimed to identify the most frequent multidrug resistance profiles of the main ten bacteria implicated in UTI acquired at the community in Aveiro District (Portugal). The results of our previous study [11] showed that (1) from the main ten bacteria implicated in UTI (*Escherichia coli, Staphylococcus aureus, Proteus mirabilis, Klebsiella spp., Enterococcus faecalis, Proteus vulgaris, Pseudomonas aeruginosa, Enterobacter spp., Staphylococcus epidermidis,* and* Providencia spp.*),* E. coli* was implicated in more than 50% of the UTI; (2) the UTI was more frequent in women (78.5%) and its incidence increased with age; (3) the resistance to antibiotics of the bacteria most implicated in UTI,* E. coli*, was lower than those of the other pathogens implicated in UTI; and (4) the bacteria isolated from males were less resistant than those isolated from females and this difference increased with the patient age. The identification of the MDR profiles in these bacteria most implicated in UTI is essential for health professionals to improve and establish an appropriate empirical therapy.

## 2. Methods

### 2.1. Study Period and Urine Sampling

In this retrospective study, all urine samples from patients of Aveiro District presenting clinical symptoms of urinary tract infection treated in ambulatory regime, collected at the Clinical Analysis Laboratory Avelab (Aveiro, Portugal) during the period 2000–2009, were included as described by Linhares et al. [11]. For each patient the age, sex, urine culture results, identification of the bacterial strain responsible for UTI, and the correspondent Antimicrobial Susceptibility Test (AST) results were registered. The Ethical Committee of the Clinical Analysis Laboratory Avelab approved this study.

The midstream clean-catch technique was used to collect the early urine sample but, for children up to two years, the urine sample was collected using a collection bag as described by Linhares et al. [11]. The urine samples were analyzed within one hour after collection. When this procedure was not possible the urine samples were stored at 4°C and processed until 24 h after collection.

### 2.2. Microscopic Examination

The urine samples were homogenized and concentrated by centrifugation and the pellet was directly examined or stained by the Gram technique as described by Linhares et al. [11].

### 2.3. Urine Culture

The urine samples were inoculated in agar plates using the streak plate method. Specific and differential culture media were used to isolate and identify the bacteria [11]. The samples were classified according to Linhares et al. [11] as contaminated when polymorphic bacterial growth was observed (exclusion criterion); as negative when bacterial growth was lower than 10^3^ cfu mL^−1^ (exclusion criterion); and as positive (inclusion criterion) when monomorphic bacterial growth was higher than 10^5^ cfu mL^−1^. For these cases and for urine cultures with monomorphic bacterial growth between 10^4^ and 10^5^ cfu mL^−1^ the AST was performed.

### 2.4. Identification of Bacterial Isolates

The bacterial strains studied in this work were isolated and identified previously as described by Linhares et al. [11]. Biochemical tests were selected based on the morphology of the isolated bacteria and on the results of the microscopic examination of the Gram-stained smear. Briefly, the Enterobacteriaceae were differentiated using the Kligler (BD BBL, 211317), Tryptone (BD BBL, 264410), Simmons Citrate (BD BBL, 211620), and Urea (Oxoid, CM0053) media. To differentiate between species of the same genus some biochemical tests were done [11].

### 2.5. Antimicrobial Susceptibility Test

The modified Kirby-Bauer disk diffusion method was used to perform the AST [11]. Briefly, a bacterial suspension in physiological saline solution was spread platted on Mueller-Hinton Agar. Antimicrobial-impregnated disks (BD BBL, Sensi-Disc) were placed on the cultures medium surface. For the Enterobacteriaceae, the antibiotics amoxicillin, cephradine, cefuroxime, amoxicillin-clavulanic acid (AMC), amikacin, gentamicin, ciprofloxacin, trimethoprim-sulfamethoxazole (SXT), and nitrofurantoin were tested. For* Enterococcus spp.* and* Streptococcus spp.*, penicillin, imipenem, amoxicillin-clavulanic acid, gentamicin, ciprofloxacin, nitrofurantoin, and vancomycin were used. For* Staphylococcus spp.*, penicillin, cephradine, amoxicillin-clavulanic acid, gentamicin, ciprofloxacin, nitrofurantoin, and vancomycin were used. For* Pseudomonas spp.*, piperacillin, cefepime, aztreonam, imipenem, amikacin, gentamicin, and ciprofloxacin were tested. After plates incubation, at 37°C for 18–24 hours, the antibiotic efficacy was determined by measuring the diameter of the zones of inhibition [12]. Bacterial strains were classified as susceptible (S), intermediate (I), or resistant (R) according the diameter of the inhibition zone [12].

### 2.6. Statistical Analysis

The Statistical Package for the Social Sciences (SPSS) 16.0 for Windows was used to analyze data. A *ρ* value of ≤ 0.05 was considered significant. The normality of data, homogeneity, and independence of variance were checked before analysis. As most of the variables failed these statistical method assumptions, the nonparametric Mann-Whitney* U *and Kruskal-Wallis tests were used. To analyze the bacterial multidrug resistance (MDR) patterns of the main ten bacteria implicated in UTI, only the antibiotics that were used in more than 85% of the cases were included. The uropathogens resistant to three or more antimicrobial classes were considered MDR [13]. To simplify the statistical treatment, only the MDR profiles observed, at least, in 5% of the MDR bacteria were selected.

## 3. Results

### 3.1. Multidrug Resistance of the Main Bacteria

**Figure 1:**
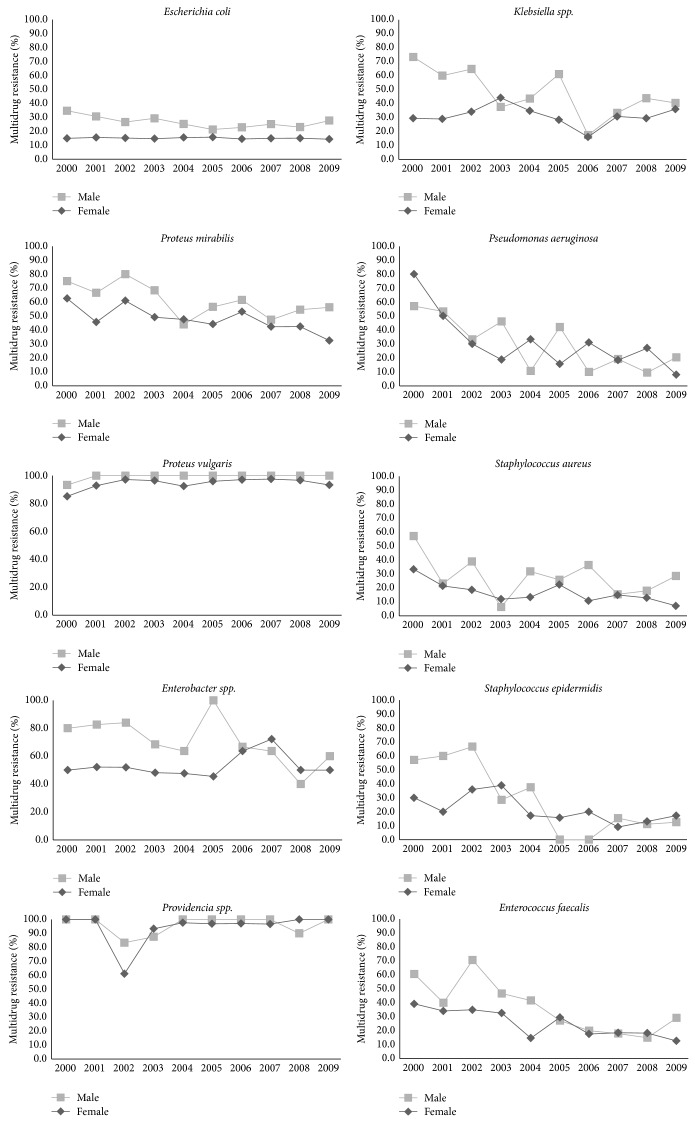
Incidence of MDR isolates of the main bacteria implicated in UTI by sex. The incidence of MDR isolates of the main ten bacteria implicated in UTI, during the study period, was determined by female and male patients.

The main ten bacteria were implicated in 17580 UTI, 4376 (25%) being multidrug resistant. A higher incidence of UTI caused by MDR bacteria was observed among male patients (35.4%) than among female patients (22.1%). The incidence of* E. coli* and* P. vulgaris* MDR isolates was higher in male patients during all the study period and the incidence of MDR isolates of* Klebsiella spp*.,* P. aeruginosa, P. mirabilis, Enterobacter spp., S. aureus,* and* E. faecalis was* higher in male patients in half of the study period (Figure 1).

A higher percentage of MDR isolates was also observed among elderly patients (30.2%).

### 3.2. Prevalence of MDR Profiles

**Figure 2:**
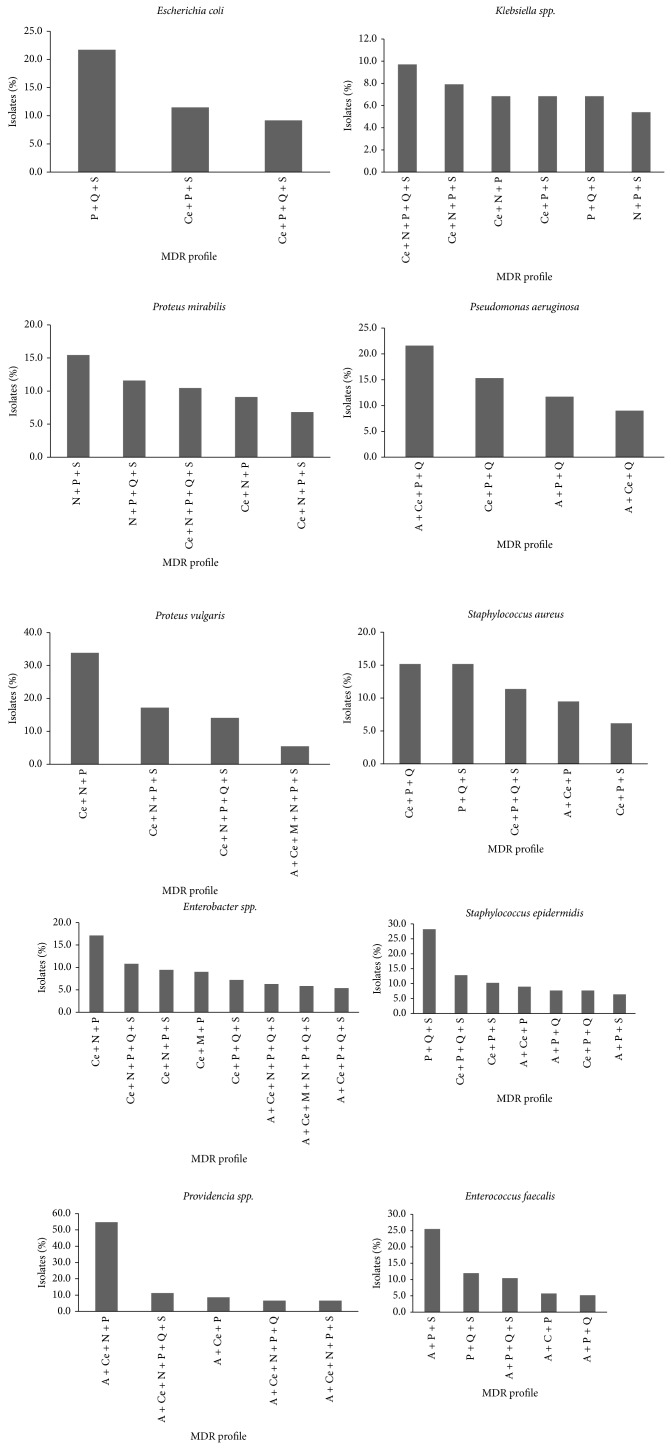
Variation of the MDR profiles in the main bacteria implicated in UTI during the study period. Only the MDR profiles in which incidence was higher than 5% in the MDR bacteria were included. P: penicillins; Q: quinolones; S: sulfonamides; Ce: cephalosporins; N: nitrofurans; A: aminoglycosides; M: monobactams.

The most common MDR profile (among those presenting an incidence higher than 5%) includes resistance to penicillins, quinolones, and sulfonamides (PQS) and was found in five of the main ten bacteria implicated in UTI (responsible for 80.2% of all UTI), namely, in* E. coli* (21.7%),* S. aureus* (15.2%),* Klebsiella spp.* (6.8%),* E. faecalis* (12%), and* S. epidermidis* (28.2%) (Figure 2). For the other bacteria, with exception of* P. aeruginosa* (for which sulphonamides are not used), this MDR profile was also observed but associated with resistance to other antibiotic classes.

The three types of MDR profiles presented in* E. coli* isolates (resistance (1) to “PQS”; (2) to cephalosporins, penicillins, and sulfonamides (CPS); and (3) to cephalosporins, penicillins, quinolones, and sulfonamides (CPQS)) were also found in* S. aureus, S. epidermidis,* and* Providencia spp.* (Figure 2). The bacterium* Enterobacter spp.*, with eight MDR profiles (all including resistance to penicillins and cephalosporins), showed the higher diversity when compared with other Enterobacteriaceae such as* E. coli* that showed the lowest diversity, three MDR profiles.

The six MDR profiles most frequent in the ten main bacteria isolated from UTI patients were observed in 43.4% of MDR bacteria and all included resistance to the penicillins class. The quinolones were the less frequent in these MDR profiles being, however, present in 50% of the 6 MDR profiles (Table 1).

**Figure 3:**
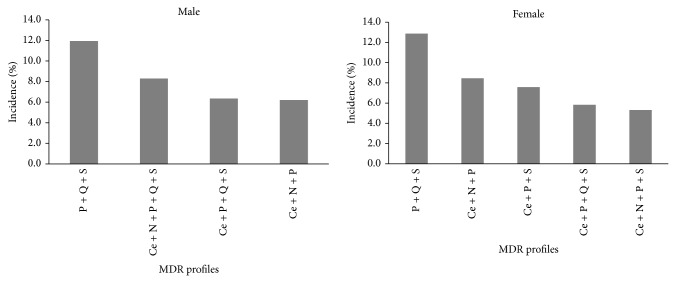
Incidence of the most frequent MDR profiles by sex. Only the MDR profiles in which incidence was higher than 5% in the MDR bacteria were included. P: penicillins; Q: quinolones; S: sulfonamides; Ce: cephalosporins; N: nitrofurans.

The most common MDR profile observed for the main bacteria implicated in UTI, which includes resistance to “PQS,” was the same observed for the bacteria isolated from male and female patients (Figure 3), but the incidence of this MDR profile was higher in bacteria isolated from female patients (12.9%) than from male (11.9%) ones. The second MDR profile (8.3%) more frequent in bacteria isolated from male patients includes resistance to five different antimicrobial classes, namely, resistance to cephalosporins, nitrofurans, penicillins, quinolones, and sulfonamides (CNPQS) (Figure 3), whereas the second MDR profile (8.5%) among bacteria isolated from female patients showed resistance to three different antimicrobial classes (cephalosporins, nitrofurans, and penicillins (CNP)).

**Figure 4:**
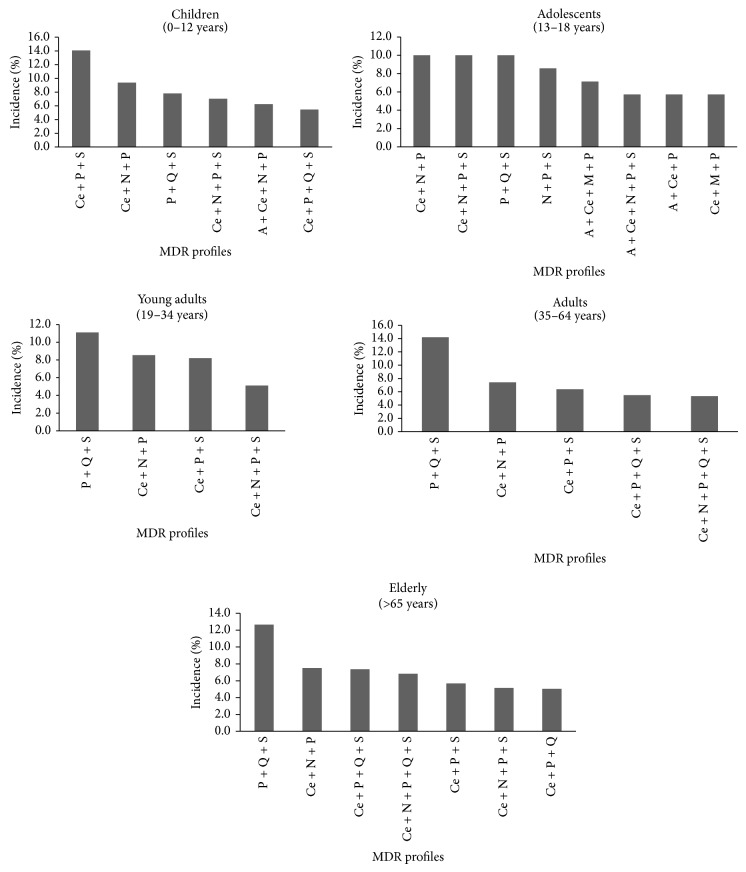
Incidence of the most frequent MDR profiles by age groups. Only the MDR profiles in which incidence was higher than 5% in the MDR bacteria were included. P: penicillins; Q: quinolones; S: sulfonamides; Ce: cephalosporins; N: nitrofurans; A: aminoglycosides; M: monobactams.

The MDR profile more common (which includes resistance to “PQS”) in the main bacteria implicated in UTI was the same for bacteria isolated from adolescents, young adults, adults, and elderly patients and its incidence increased with the age of the patients. Contrarily, in bacteria isolated from children, this MDR profile was the third most frequent profile (7.8%), and for this group the most frequent MDR profile included resistance to “CPS” (14.1%) (Figure 4). A high diversity of different MDR profiles was observed for bacteria isolated from adolescents (8 profiles). The lowest number of MDR profiles was observed for bacteria isolated from young adults for whom only 4 different MDR profiles were observed (Figure 4).

**Figure 5:**
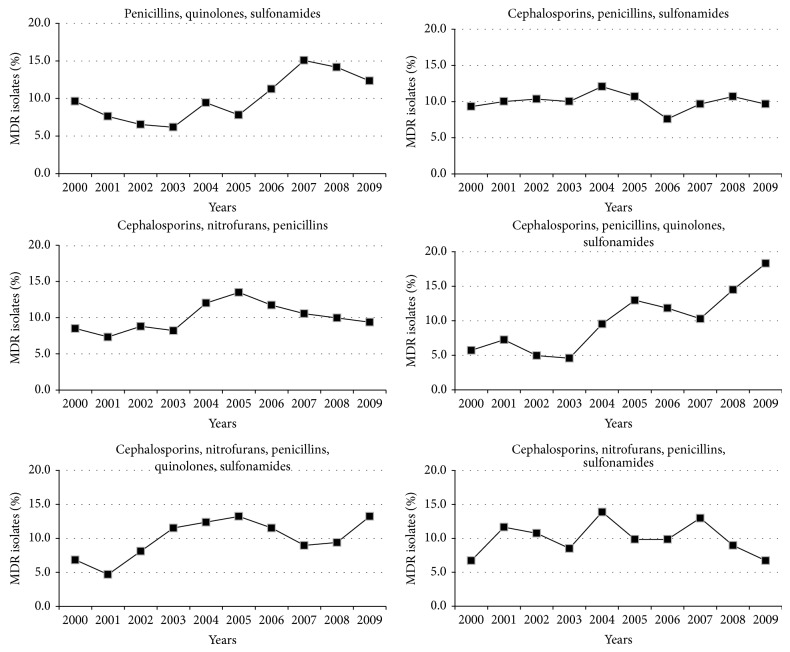
Incidence of the six most frequent MDR profiles during the study period. Only the MDR profiles in which incidence was higher than 5% in the MDR bacteria were included.

The incidence of 3 most frequent MDR profiles (resistance to (1) “CPS”; (2) “CNP”; and (3) cephalosporins, nitrofurans, penicillins, and sulfonamides (CNPS)) was stable during the study period (Figure 5); however, the incidence of the other 3 most frequent MDR profiles changed over the study period, namely, those that confer resistance to “CPQS” (increase of 12.6%); to “CNPQS” (increase of 6.4%); and to “PQS” (increase of 2.7%) (Figure 5).

## 4. Discussion

Multidrug resistance was observed among the most prevalent bacteria involved in the community-acquired UTI, a quarter of these bacteria being resistant to three or more antimicrobials of distinct classes, and the most incident MDR profile includes resistance to “PQS.”

The most common MDR profile (resistance to “PQS”) was found in five of the main ten bacteria implicated in UTI (*E. coli*,* S. aureus*,* Klebsiella spp.*,* E. faecalis,* and* S. epidermidis*) but was also observed in the other five bacteria implicated in UTI which present simultaneously resistance to other antibiotic classes. As sulfonamides are not used for* P. aeruginosa* this MDR profile was not observed for this bacterium. This MDR profile includes most of the antibiotics recommended by the European Association of Urology for empirical therapy of uncomplicated UTI which suggest that the UTI standard empirical therapy treatment guidelines must be adjusted to the community of Aveiro District. Moreover, this MDR profile includes antibiotics with three different mechanisms of action which limits the therapeutic options available to treat UTI.

Few similar studies, performed at community level, are available for the main implicated bacteria in UTI, which makes comparison difficult of the prevalence of the MDR profiles observed in this community with those found in other locations. However, for the most implicated bacterium in UTI,* E. coli*, similar studies showed that ciprofloxacin-resistant* E. coli* isolates were simultaneously resistant to ampicillin and to SXT [14]. Another study also showed that fluoroquinolone-resistant strains of* E. coli* were resistant to AMC and to SXT [15].

The presence of penicillins, quinolones, and sulfonamides in the most common MDR profile would be associated with the high prescription of these antimicrobials classes in Aveiro community between 2000 and 2009. This relationship was observed for two of these antimicrobial classes, penicillins and quinolones, but not for the sulfonamides.

The penicillins class was included in the most frequent MDR profile and also in the other 5 MDR profiles more frequently among the ten main bacteria. The high administration of penicillins in Portugal at community level between 2000 and 2009 [16, 17] and the ESBL-production among the main bacteria involved in UTI [18] may explain the low efficacy of penicillins in the treatment of these infections.

The quinolones, although included in the most common MDR profile, were the antimicrobial class less frequent in the 6 MDR profiles observed in the main ten bacteria implicated in UTI but were yet present in 50% of the 6 MDR profiles. Portugal continues to be the third country among the European countries with the highest quinolones consumption which may explain the presence of this antimicrobial class in the most common MDR profile [16, 17].

The sulfonamides were observed in 83% of the main MDR profiles among the bacteria more implicated in UTI. However, contrarily to penicillins and quinolones, the sulfonamides were the antimicrobial less prescribed between 2000 and 2009 in Portugal [16], meaning that their presence in the most frequent MDR profiles is not associated with their prescription. Some studies showed that the decrease of antibiotics consumption among outpatients was followed by a significant decrease of resistance to these antibiotics [19, 20]. However, a recent finding in UK contradicted these studies because despite the prescription of sulfonamides being drastically reduced and even almost abolished the resistance to these antimicrobials by* E. coli* remained high [21]. These results, as well as those obtained in the present study, may be explained by the often close link of sulfonamide-resistant gene with other resistance determinants on mobile genetic elements such as plasmids that also include genes encoding resistance to other antibiotics, which continued to be administered, allowing the selection of these multiresistance plasmids and consequently maintaining sulfonamide resistance [21, 22]. Another possible explanation is the fitness cost that determines the rate at which a decrease of resistance will occur in response to the reduction of antimicrobials use [23]. According to Lipsitch [23] the bacteria do not pay a high fitness cost on the transmissibility of sulfonamide resistance and even when antimicrobials pressure is removed, resistant strains remain [22]. The* sul2* is the most prevalent gene and its acquisition represents the most common mechanism that enables bacteria to maintain sulfonamide resistance over the time.

The close link of antibiotics-resistant gene, with other resistance determinants on mobile genetic elements and probably the low fitness cost to maintain antimicrobial resistance, can also explain the highest diversity of MDR profiles observed for* Enterobacter* isolates. Zervos et al. [24] observed in* Enterobacter* sp. isolates the presence of three integron-mediated resistance sets that can be maintained without biological cost, allowing, by this way, the maintenance of resistant bacterial strains even when the antibiotic selective pressure is reduced [25].

The presence of quinolones in 50% of the most common MDR profiles and the high resistance among the main bacteria implicated in community-acquired UTI may be explained by the high consumption levels. Studies performed at community level [24] showed that a huge decrease of quinolones prescription may contribute to diminishing the prevalence of resistance to this antimicrobial class since bacteria pay a high fitness cost on the transmissibility of quinolone resistance [22].

Although only a slight increase (about 2.7%) in the incidence of the six most common MDR profiles was observed during the study period, the MDR profiles that include simultaneously resistance to quinolones and cephalosporins (corresponding to the fourth and fifth more frequent MDR profiles) showed a higher increase (about 12.6 and 6.4%, resp.). The increase of resistance to cephalosporins may be related with a possible increase of ESBL-production among Enterobacteriaceae that also confers resistance to other beta-lactams antibiotics, including third- and fourth-generation cephalosporins [26]. In addition to ESBLs, other less frequent enzymes, also clinically important as plasmid-mediated AmpC (first described for* E. coli*), confer resistance to most of the penicillins, to beta-lactamase inhibitor plus beta-lactam combinations, and to first- (cefazolin and cefalotin) and second-generation cephalosporins (cefoxitin) [27]. According to the literature, quinolone resistance is associated with ESBL-production, being the production of CTX-M, enzymes common among bacteria implicated in community-acquired UTI, namely, Enterobacteriaceae such as* E. coli*, a serious cause of community-acquired UTI worldwide [28]. Several studies showed that genes conferring plasmid-mediated quinolone resistance have been associated with blaCTX-M genes that typically enable hydrolyze of third-generation cephalosporins, such as cefotaxime, more efficiently than to ceftazidime [29]. High frequency of quinolone resistance was found in ESBL-producing* E. coli* (70%) [30]. This association can explain the increase in the incidence of MDR profiles including these two antimicrobials along the study period.

The incidence of the most common MDR profile was higher in MDR bacteria isolated from female patients and its incidence increased with the age of the patients. Most of the UTI in men are complicated, being associated with structural or functional abnormality in urinary tract, requiring frequently prolonged antimicrobial therapy, and needing antimicrobials that can reach high therapeutic concentrations in the prostatic tissues, namely, drugs that are able to cross the electrically charged lipid membrane of the prostate epithelium [31]. Probably for these reasons, male patients are more likely to develop resistance to antibiotics. The increase of the incidence of the most common MDR profile with the age of the patients may be explained by the increase of the number and duration of hospital admissions and due to the immune system fragility of the elderly patients. The incidence of the most common MDR profile was higher when bacteria were isolated from young adults, adults, and elderly patients; however, the same was not observed for MDR bacteria isolated from children. The absence of quinolones in the most frequent MDR profile (including resistance to cephalosporins, penicillins, and sulfonamides) observed for bacteria isolated from children may be explained by the restriction of these antimicrobials in paediatric patients due to the potential of adverse cartilaginous effects [32]. Nevertheless, the quinolones prescription for paediatric patients has increased during the last years, their use being recommended by the American Academy of Pediatrics in specific clinical settings, including urinary tract infections caused by Gram-negative bacteria,* P. aeruginosa*, or other multidrug resistant bacteria [32]. Despite these evidences, the MDR profile, including “PQS,” was the third MDR profile more observed among MDR bacteria isolated from children, which according to Charalabopoulos et al. [31] may be due to person-to-person transmission between families, in day care or school settings, and even the use of fluoroquinolones in poultry populations [29].

## 5. Conclusion

As (1) the most common MDR profile observed in the main ten bacteria includes antibiotics with three different mechanisms of action; (2) the most common MDR profile includes the main antibiotics recommended by the European Association of Urology for empirical therapy of uncomplicated UTI; (3) two of the three antibiotics (penicillin and quinolones) included in the most incident MDR profile and also present in more than 50% of the six more frequent MDR profiles were regularly prescribed during the study period; and (4) five of the six more frequent MDR profiles, comprising the most common MDR profile, include resistance to an antibiotic (sulfonamides) that during the study period was avoided and lately even abolished, the results of this study suggest that (1) the high percentage of MDR bacteria (25%) and the high diversity of MDR profiles (6 profiles with an incidence higher than 5%) result from a high prescription of antibiotics but also from antibiotic-resistant genes that are transmitted with other resistance determinants on mobile genetic elements and (2) the UTI standard treatment guidelines must be adjusted for the community of Aveiro District.

## Figures and Tables

**Table 1:** Percentage of the most common MDR profiles (incidence higher than 5% in MDR bacteria).

MDR profiles	Number of MDR isolates	% MDR isolates
Penicillins, quinolones, and sulfonamides	551	12.6
Cephalosporins, nitrofurans, and penicillins	341	7.8
Cephalosporins, penicillins, and sulfonamides	290	6.6
Cephalosporins, penicillins, quinolones, and sulfonamides	262	6.0
Cephalosporins, nitrofurans, penicillins, quinolones, and sulfonamides	234	5.3
Cephalosporins, nitrofurans, penicillins, and sulfonamides	223	5.1
